# Lotus Leaf-Inspired Corrosion-Resistant and Robust Superhydrophobic Coating for Oil–Water Separation

**DOI:** 10.3390/biomimetics10050262

**Published:** 2025-04-24

**Authors:** Wenhui Tu, Yiwen Luo, Junhao Shen, Xu Ran, Zhe Yu, Chaolun Wang, Chunhua Cai, Hengchang Bi

**Affiliations:** 1 In Situ Devices Center, School of Integrated Circuit, East China Normal University, Shanghai 200241, China; 2 Chongqing Key Laboratory of Precision Optics, Chongqing Institute of East China Normal University, Chongqing 401120, China

**Keywords:** biomimetics, spraying, superhydrophobic coating membrane, oil/water separation, durability

## Abstract

With daily oil consumption approaching 100 million barrels, the global demand continues to generate significant quantities of oily wastewater during oil extraction, refining, and transportation, and the development of effective oil–water separation technologies has become crucial. However, membrane corrosion is a challenge under the harsh conditions involved. Here, we are inspired by the lotus leaf to create a corrosion-resistant and robust superhydrophobic membrane using a general spraying method. By using this spraying process to apply the Graphene@PDMS heptane dispersion onto the mesh substrate, we create a biomimetic corrosion-resistant and robust superhydrophobic stainless steel mesh (SSM). The modified SSM can still maintain superhydrophobic properties after soaking in a strong acidity solution (pH = 1), robust alkalinity solution (pH = 14), or NaCl solution (15 days), which demonstrates excellent chemical stability. Moreover, the modified SSM shows strong mechanical stability during ultrasonic treatment for 2 h. The superhydrophobic SSM can be used to separate various kinds of oils from water with high flux and separation efficiency. It shows a high flux of 27,400 L·m^−2^·h^−1^ and high separation efficiency of 99.42% for soybean oil–water separation using 400-mesh SSM. The biomimetic modified SSM demonstrates great potential for oil–water separation under harsh conditions, which gives it promise as a candidate in practical applications of oil–water separation.

## 1. Introduction

The problem of crude oil leakage caused by natural disasters, like volcanic eruptions and earthquakes, has not only seriously threatened the survival of marine animals and plants, but also brought significant economic losses to relevant regions. Furthermore, global oil consumption approaches 100 million barrels per day, and significant quantities of oily wastewater are produced during the extraction, refining, and transportation of oil [1]. The World Bank cautions that ineffective management of oily wastewater may result in a 6% reduction in GDP growth rates and could incur a loss of USD 4.5 trillion by 2050 [2], making it urgent and necessary to develop effective oil–water separation techniques. Conventionally, various approaches including in situ burning, skimmers, biodegradation, and sorption have been developed and employed for treating oil–water mixtures [3,4,5,6]. These techniques show the advantages of timeliness, environmental compatibility, and extensive specific surface areas. However, considerations such as operational costs and limited processing capacities limits their practical application. Recently, innovative technologies for the treatment of oily wastewater have emerged, utilizing membranes [7] such as nanofibers [8,9], meshes [10,11], and cellulose [12], which exhibit unique wetting properties like hydrophilicity and oleophilicity. Among them, superhydrophobic membranes allow oil to pass through under the weight of gravity, while blocking water. These technologies also feature self-cleaning and anti-corrosion properties [13], making them suitable for authentic applications in corrosive or other harsh conditions environments.

Despite the progress made in superhydrophobic coatings, there are still challenges to overcome. The durability and stability of these coatings under harsh environments, as well as their adhesion to the metal substrate, are important factors that need to be resolved for effective industrial application. Various materials, including nanowire composites [14,15,16,17,18], and chemically inert inorganics [19,20,21,22,23,24], have been explored for their potential in enhancing superhydrophobic surfaces. Furthermore, Hashjin et al. [25] discovered that by adjusting the pH of the sols and alternately applying them on a glass substrate, they could significantly improve the robustness of the superhydrophobic coating using a sol–gel process. Similarly, Ju et al. [26] utilized micro-skeleton protection [21,27] to develop a robust superhydrophobic surface. This involved creating a composite with a rigid micro-skeleton of chromium (Cr) microparticles within an epoxy layer, then filling the structure with stearic acid-modified copper (Cu) powders, and optimizing the process for enhanced mechanical and chemical stability as well as anti-icing/frosting properties. However, these approaches frequently encounter issues such as high energy usage, secondary contamination, or intricate manufacturing procedures [28].

**Scheme 1:**
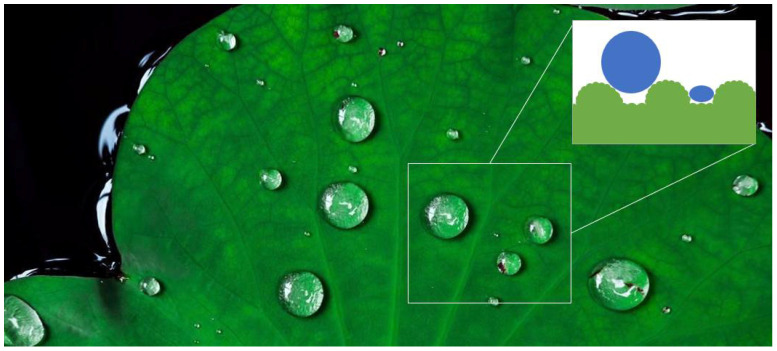
Biomimetic lotus leaf structure demonstrating its natural superhydrophobicity.

In recent years, graphene and its derivatives have attracted considerable attention in the field of water treatment, especially for oil–water separation. This interest is due to their carbon atom skeletons with hydrophobic and lipophilic groups [29] and other exceptional properties [30,31,32,33]. Polydimethylsiloxane (PDMS) is extensively utilized because of its low toxicity, transparency, chemical stability, and minimal surface energy [34,35,36,37]. Among membrane materials, metal with high ductility, excellent thermal stability, and extended service life is rather suitable for harsh application environments. Among various porous metal materials, stainless steel mesh is chosen as the primary framework due to its benefits, including abrasion resistance and high compressive strength. Meanwhile, the flourishing advances in biomimetics have provided new insights into the research and development of functional materials and devices [38,39,40,41], such as achieving superhydrophobicity through lotus leaf emulation. Recent studies have further revealed that dynamic interactions between droplets and superhydrophobic surfaces (e.g., rotating-induced doughnut-shaped bouncing with reduced contact time) can significantly enhance liquid repellency performance [42]. As shown in Scheme 1, the surface of lotus leaf has a hierarchical micro/nanostructure to provide stable and durable hydrophobicity, effectively reducing water adhesion even under dynamic conditions. Inspired by this, we aim to integrate nature’s robust water-repellent mechanism with advanced material science, achieving a highly stable and corrosion-resistant superhydrophobic surface, ensuring long-term durability in complex environments. While current studies have explored superhydrophobic materials for diverse applications (e.g., self-cleaning windows, oil–water separation, and even flame-retardant transparent structures) [43], our work focuses on optimizing industrial-scale durability and separation efficiency.

Herein, we aim to achieve a biomimetic corrosion-resistant superhydrophobic coating on stainless steel mesh substrates in a simple and efficient approach. We proposed a rapid one-step spray method employing a bio-inspired Graphene@PDMS dispersion to prepare corrosion-resistant and robust superhydrophobic coatings. By simulating the hierarchical roughness of lotus leaves, PDMS acts as both a low surface energy modifier and crosslinking agent, while graphene further contributes to the micro/nanostructure construction, thereby enhancing surface roughness and hydrophobic stability. The modified surface exhibits excellent superhydrophobicity for droplets with different pH levels in static angle measurement as well as dynamic rolling and impact experiments. Additionally, we evaluate the durability and adhesion of the coatings under various corrosive or destructive conditions, finding that they maintain superhydrophobic after sonication for 2 h. The modified SSM inherits the advantages of biomimetic structural design and possess high flux and separation efficiency varying with the pore sizes. Typically, the modified 400-mesh SSM can separate soybean oil from water with flux of 27,400 L·m^−2^·h^−1^ and separation efficiency of 99.42%. Moreover, the coatings can be successfully applied to both organic and inorganic substrates, extending applications.

## 2. Materials and Methods

### 2.1. Preparation of GO and Graphene

GO was prepared by oxidizing expandable graphite powders using the modified Hummers method (Bi et al. [44]). The procedure involved mixing graphite flakes (2 g, purity > 99.7%; Qingdao Chemical Reagent Co., Ltd., Qingdao, China) and sodium nitrate (1 g) with 46 mL of 98 wt% sulfuric acid in an ice bath. Potassium permanganate (6 g) was added slowly and reacted at 35 ± 1 °C for 8 h. The mixture was then diluted with 92 mL of deionized water and maintained at 98 °C for 15 min to enhance oxidation. After centrifugation, the product was washed multiple times with 5% hydrochloric acid and distilled water, followed by overnight drying at 60 °C.

The preparation of graphene was as follows: graphene powder was prepared by heating GO at 800 °C in an argon atmosphere for 3 h.

### 2.2. Preparation of PDMS@Graphene Coating

Preparation of Graphene–PDMS Heptane Dispersion: To prepare the dispersion, graphene powder (2 g) was added into 40 g/L of Sylgard 184 PDMS (Dow Corning, Midland, MI, USA, base agent:curing agent = 10:1 by weight) heptane dispersion (1 L). The PDMS was selected for its low toxicity, chemical stability, and ability to form transparent elastic films after curing at 25–150 °C without byproducts. The mixture was then dispersed using ultrasonic treatment at a power of 100 W for 60 min, resulting in a PDMS@Graphene heptane dispersing solution.

Preparation of PDMS@Graphene Coating: After transfering the PDMS@Graphene heptane dispersion into a spray gun, it may be sprayed evenly onto various substrates, such as copper mesh, SSM, nylon cloth, paper towel, weighing paper, velveteen spray cloth, and printing paper. Inspired by the hierarchical micro/nanostructure of lotus leaves, the spray-coating process was designed to construct a rough surface, mimicking nature’s stable superhydrophobic properties. This bio-inspired strategy enhances the coating’s durability and water-repellent performance, ensuring its stability under various conditions. Finally, dry the samples in an oven at 80 °C for 30 min.

### 2.3. Dynamic Droplet Adhesion Test and Oil/Water Separation

Dynamic droplet adhesion test: A 400-mesh SSM was tilted at a certain angle, and methylene blue staining drops were dropped onto the upper end of mesh to observe water droplet adhesion on the surface. This test was conducted on the mesh both before and after modification. Moreover, the modified 400-mesh SSM was placed horizontally, and deionized water droplets, salt droplets (1 wt%), strong acid (1 M), and strong alkali droplets (1 M) were dropped at a certain height above it, respectively. At the same time, the camera captured and recorded the behavior of the drops on the surface. SSM info: SUS304 stainless steel from Shanghai Yanjin silk screen processing factory.

Oil/water separation: Five types of oils and organic solvents were used in this study, including diesel oil, engine oil, soybean oil, pump oil, and heptane. SSM with mesh sizes of 300, 600, 800, and 1000 were employed. In addition, the modified 400-mesh stainless steel strip net was placed over the opening of the two beakers, maintaining a certain distance between them. Water was dyed with methylene blue, while heptane was stained with Sudan red, and these solutions were combined to form an oil/water mixture. The mixture (50%, *v*/*v*) was poured onto the mesh at the mouth of one of the beakers. The hierarchical roughness of the bio-inspired coating ensures selective permeability, allowing oil to pass while repelling water efficiently. The oil (upper layer) and water (lower layer) sequentially passed through the mesh were collected in the two beakers by gravity. Separation efficiency is calculated by *m*1/*m*0. The separation efficiency was assessed using the formula, where *m*0 represents the initial mass of the liquid before separation, and *m*1 is the remaining mass after separation. The membrane’s dynamic flux performance (DFP) was evaluated using a filtration device at ambient temperature in an open environment and calculated with Equation (1):

(1) DFP=ΔVA·ΔT

where *A* is the effective filtration area of the membrane, and Δ*V* is the volume of filtrate within the preset time of Δ*T*.

### 2.4. Coating Stability and Firmness Tests

The stability of the coating was tested by recording the contact angle of the coating immersed in solutions with pH = 3, 5, 7, 9, 11, 13 for one week and in the 5 wt% NaCl solution for various durations (total 15 days). To test the firmness of the coating, the modified SSM was placed in an ultrasonic environment (40 kHz, 100 W). It was taken out at regular intervals (20 min) to record the contact angle of the current coating. Inspired by the robust micro/nanostructures found in nature, the bio-inspired coating exhibits strong adhesion to the substrate, maintaining stability even after extended ultrasonic treatment.

### 2.5. Characterization

The surface morphology of the prepared materials was examined by scanning electron microscope (SEM). The SL200KB instrument (American KeNuo Industrial Co., Ltd., New York, NY, USA) was used to measure the contact angle of water at ambient temperature. In all measurements, the volume of individual water and oil drops is 10 μL. Repeat the measurement of the same sample taken at the same position based on at least five photos to obtain the average contact angle of water.

## 3. Results and Discussion

**Figure 1:**
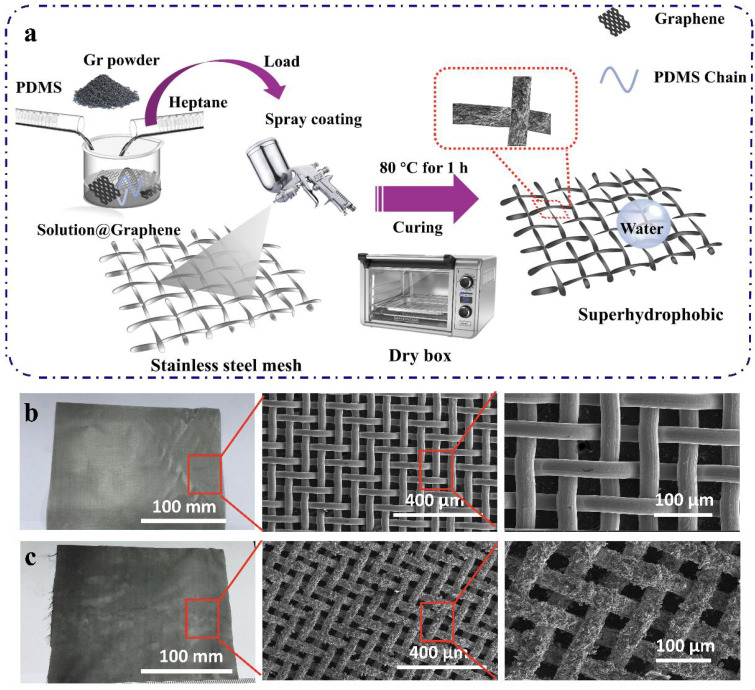
Preparation and characterization of coatings. (**a**) Fabrication of the superhydrophobic coating. SEM images of (**b**) the OSSM and (**c**) the as-prepared PDMS@Graphene coating surface at low and high magnifications.

The spray-coating method can be utilized to create coatings for different substrates. SSM, commonly used in our daily life, is chosen for its good stability and soft texture, which allows for easy cutting and bending. Therefore, stainless steel mesh is selected as the skeleton for fabricating superhydrophobic coating in this study. Initially, a diluent solution of PDMS was prepared by mixing heptane and PDMS in a certain proportion. Graphene, reduced from graphene oxide, was incorporated into the PDMS solution, thoroughly mixed using a magnetic stirrer, and then dispersed via ultrasonication for 60 min. The obtained uniform solution was sprayed onto the surfaces of various substrates and then placed in a drying oven for curing later. Figure 1a illustrates the general process of modification of 400-mesh SSM by the one-step spraying method and qualitatively demonstrates the influence of modification on the substrate surface from both macro and micro perspectives.

The surface morphology of both the original and coated substrates was characterized by SEM. Figure 1b shows the photos and SEM images of the original stainless steel mesh (OSSM) with an aperture size of 38 μm (400-mesh). The SEM images show that the OSSM surface is smooth. After coating with the prepared mixture, the fluffy, coral-like graphene attaches tightly to the substrate surface, leading to a slight decrease in the pore size of the mesh (Figure 1c). The SEM image at high magnification in Figure 1c displays the random micro-scale clusters of graphene. As described in Figure S1, we have successfully applied the same fabrication approach to various materials, including nylon (Figure S2), tissue (Figure S3), non-woven fabric (Figure S5), and copper mesh (Figure S6), to create superhydrophobic coatings.

**Figure 2:**
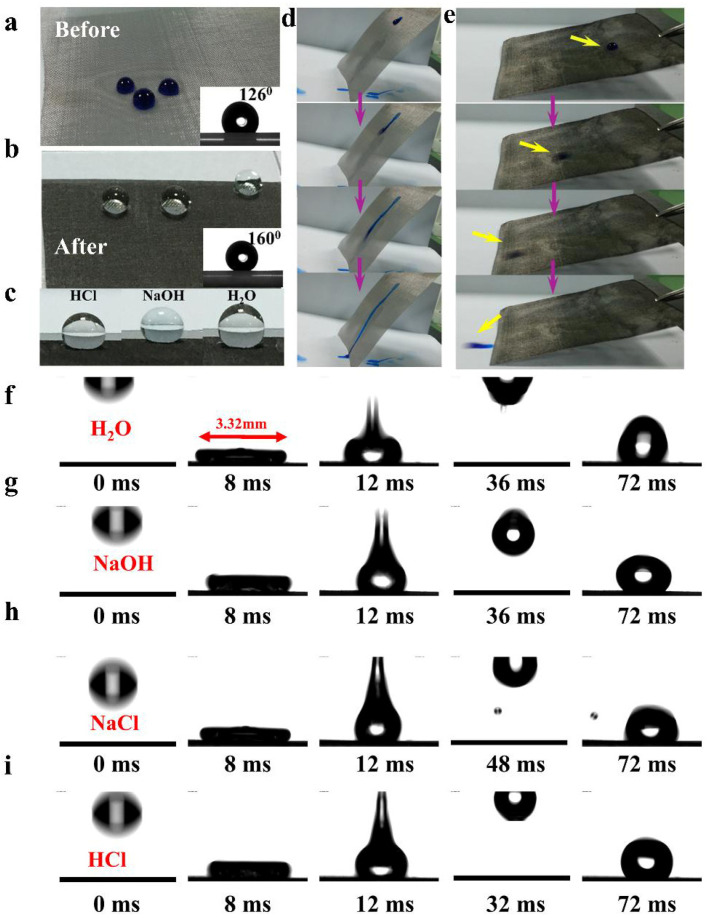
Wetting behavior of the mesh is depicted (**a**) before and (**b**) after coating, toward droplets with different pH. Their insets show the contact angles of the water droplet. (**c**) Side view of strong acid, strong alkali, and water droplets on modified 400-mesh stainless steel mesh. Droplet adhesion test on mesh surface (**d**) before and (**e**) after coating. Dynamic droplet impact experiments using four different kinds of liquid, H_2_O (**f**), NaOH (**g**), NaCl (**h**), and HCl (**i**), respectively.

The roughness of modified SSM and its affinity for water or oil are crucial for its superhydrophobic properties in the air. The wettability of a solid surface is dependant on its chemical composition and physical structure [45,46,47,48]. The low surface free energy of PDMS we use is consistent with superhydrophobic wettability [49,50]. Graphene has a broad absorption capacity for crude oil, alkanes, toluene, and other organic solvents due to its excellent hydrophobicity and lipophilicity [51,52]. In the three-phase contact system formed by solid, liquid, and gas, some gas is trapped under the droplet, which promotes the formation of the Cassie–Baxter state [53]. The contact angle is used to evaluate the wettability of the mesh surface. To enhance the visual effect and facilitate observation, the droplets are dyed. In the air, water droplets with varying pH dropped onto the surface of the unmodified mesh spread and exhibit a contact angle of ~126° (Figure 2a). In contrast, droplets on the modified mesh surface remain relatively undisturbed, showing a contact angle of around 160° (Figure 2b). This demonstrates that the hydrophobic coating of the modified stainless steel mesh (MSSM) exhibits stable and distinct wettability towards corrosive solutions (1 M HCl and 1 M NaOH solutions). Inspired by the biomimetic lotus leaf structure, Figure 2c further contrasts the side view of the deionized water, strong acid, and strong alkali droplets on the MSSM surfaces. When the substrate is changed to tissue widely used for water absorption in our daily lives, this comparison becomes more intense. Refer to Figures S7–S12 (Supplementary Materials) to observe the surface-wetting behavior of tissue as well as other polymer materials and metal nets with different mesh numbers.

Understanding the behavior of liquids under static conditions is crucial for studying the surface properties of superhydrophobic materials. However, to gain a more comprehensive understanding of their performance, it is necessary to explore the behavior of liquids under dynamic conditions. Therefore, we chose two types of dynamic droplet experiments to better evaluate the superhydrophobic performance of liquids. Stainless steel mesh carrying the water droplets was tilted at a specific angle, and snapshots were taken to document the movement of the droplets on modified and original surfaces (Figure 2d,e). This further demonstrates that the superhydrophobic coating maintains its superhydrophobicity against acidic droplets, alkaline droplets, and certain saline solutions. The superhydrophobic performance of these three liquids under static conditions was further confirmed through the dynamic droplet impact experiments. This study focuses on the dynamics of droplet impingement on superhydrophobic coatings. For these tests, each droplet (10 µL) was dropped statically at a height of 5 cm, achieving an impact velocity of ~0.98 m/s upon hitting as depicted in Figure 2f, the collision of a water droplet with a superhydrophobic coating can generally be categorized into three stages: expansion, contraction, and full rebound. Taking the moment the water droplet begins to fall (marked as 0 ms), the droplet is observed spreading on the surface. At 8 ms, the droplet reaches its maximum spread diameter of 3.32 mm on the superhydrophobic surface, which is 12 times more than its original diameter. Just like the theoretical analysis, due to the significantly increased surface roughness of the treated membrane, an air layer forms on the coating surface, which obstructs the contact area between the membrane surface and the water droplet. As a result, the droplet rebounds fully without adhesion to the surface.

However, the droplet’s kinetic energy is dissipated by internal viscous drag, resulting from the adhesion force exerted by the liquid droplet. Consequently, the droplet’s energy loss accumulates over time, causing a gradual reduction in its height after each impact. This results in a less pronounced rebound in the latter stages of the process [50]. Figure 2g–i repeated the experiment under the same experimental conditions but used acid, alkali, and salt solutions instead. The maximum spreading diameters for the strong acid, strong alkali droplets, and salt droplets were measured at 2.7 mm, 2.86 mm, and 3.12 mm, respectively, showing similar values. We also did experiments with water droplets rolling on different substrates, and Movies S1 and S2 recorded the experimental procedures using stainless steel mesh and nylon cloth as skeletons, respectively. One direct explanation is that the micro/nano-structured surface of the superhydrophobic coatings can repel droplets and prevent moisture accumulation, which is a common cause of corrosion. These observed phenomena above can be attributed to the chemical properties and micro-scale roughness of PDMS and graphene used in the coatings, inspired by nature’s biomimetic structure. This extends the application of this technology, particularly in environments exposed to acid or alkaline rain resulting from modern industrial pollution.

**Figure 3:**
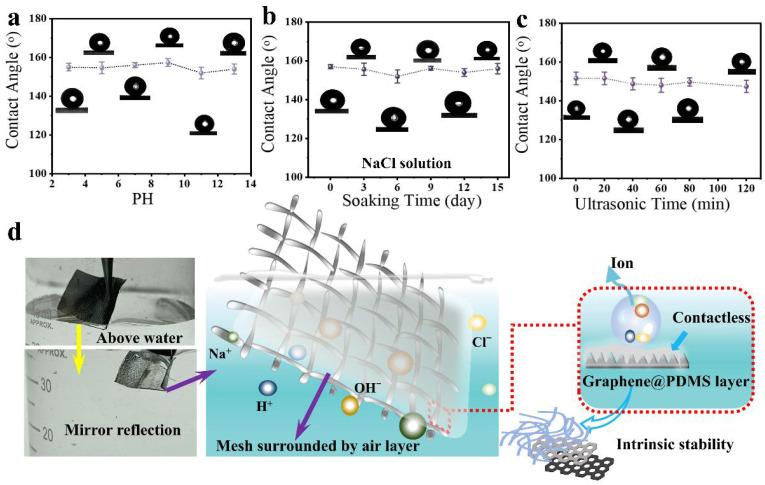
(**a**) The change in contact angle of water droplets under different pH treatments. (**b**) The change in contact angle of water droplets under saline immersion treatment. (**c**) The change in contact angle of water droplets under ultrasonic treatment. (**d**) Formation principle of the corrosion-resistant superhydrophobic surface.

Superhydrophobic surfaces are inevitably exposed to harsh environments, including excessive acidity and alkalinity or possible mechanical forces, which could damage the micro/nanostructures on the surface and decrease the super-hydrophobicity. Thus, inspired by the stable and durable natural superhydrophobicity of structures like the lotus leaf, which has evolved to withstand such harsh conditions, the resistance of harsh conditions is one of the key factors in determining the feasibility of superhydrophobic surfaces in basic research and practical application. In our study, we conducted durability tests on Graphene@PDMS superhydrophobic samples using saline, hydrochloric acid (HCl), and sodium hydroxide (NaOH) solutions. Additionally, we performed stability tests under conditions of ultrasound treatment and prolonged saline immersion. These tests were designed to evaluate how the biomimetic superhydrophobic coatings could maintain their performance under various conditions. Figure 3a shows the relationship between the pH value of the soaking solution and the CA value of water on the superhydrophobic coating after the soak test. After soaking for the same time in 14 different pH solutions (from 1 to 14), the CA value on the superhydrophobic coating remained almost unchanged within the experimental error range, indicating that the superhydrophobic coating has good chemical stability against acidic droplets, alkaline droplets, and some saline solutions.

To further validate these findings, we increased the mesh number, repeated the experiment, and observed consistent results (Figure S13). Figure 3b depicts the change in the contact angle (CA) of water on the superhydrophobic coating’s surface as the duration of immersion in a 5 wt% NaCl solution increases. Similarly, even after 15 days for immersion, the CA of the coating surface shows no significant change. When substituting stainless steel with 500-mesh copper, the water droplets continue to form a spherical shape on the coated samples, maintaining superhydrophobicity with CA > 150° (Figure S13). Figure 3c shows the changes in the contact angle of water on the superhydrophobic coating after ultrasonic treatment for different durations (0–120 min). It was observed that the CA values on the superhydrophobic coating only undergo minor changes. Light microscopy (Figure S14) revealed that the surface morphology of the durable superhydrophobic coating remained largely unchanged even after different periods of ultrasonic treatment.

From the stability test discussed, the modified coating retains its superhydrophobicity under various external influences. This unique interfacial property, inspired by nature’s resilient superhydrophobic structures, contributes to the resilience of the coating against the impact of harsh environmental conditions, as demonstrated in Figure 3d. When the corrosion-resistant and robust stainless steel mesh is submerged in water, a notable transition in the surface properties occurs, shifting from the original dull color to a reflective bright white appearance, as seen in the comparative photos above and below the water. This transformation is attributed to the superhydrophobic properties of the mesh, which facilitate the formation of an air layer on its surface, thereby creating a barrier against water contact. In addition to the inherent chemical stability of graphene as a carbon material, PDMS is also stable. Its monomers are continuously supplied to the surface to cover newly exposed stainless steel, caused by poor conditions, which should be the reason for its long-term protection of micro/nano structures [54]. All of these observations indicate that the modified superhydrophobic coating is highly durable, combining chemical and physical stabilities to make it a promising candidate for future practical applications.

**Figure 4:**
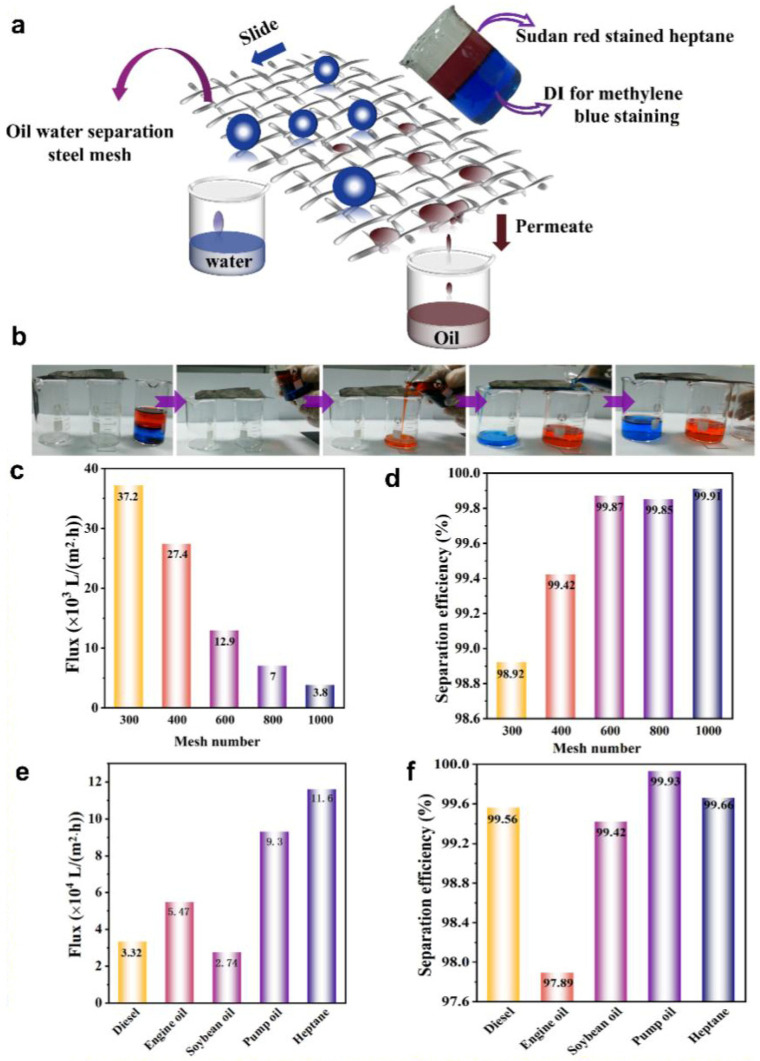
Studies on oil/water separation using Graphene@PDMS coating. (**a**) Demonstration of the oil/water separation process, (**b**) oil/water separation experiment, and the flux (**c**) and separation efficiency (**d**) of soybean oil/water mixture using MSSM across various mesh sizes. Flux (**e**) and separation efficiency (**f**) of 400-mesh MSSM for different mixtures of oil and water.

Given the contrasting wetting properties of water and oil on hydrophobic coating films, it is reasonable to expect that these two liquids would exhibit different levels of permeability through the film [55]. To demonstrate this peculiarity, we proceeded to test the separation of oil/water mixtures. The mixture of oil and water was poured onto a metal mesh placed between beakers, with the sole driving force for separation being gravity. During the pouring of the oil/water mixtures onto the metal mesh covering one of the beaker openings, water could remain stable on the mesh and flow into the next beaker following the incline trend, while oil rapidly permeated through and was collected by the beaker below. Figure 4a provides a schematic of the gravity-driven separation process for the heptane–water mixture, and Figure 4b shows the whole process of this horizontal structure oil/water separation.

To quantitatively evaluate the separation quality, an oil/water (50%, *v*/*v*) mixture was prepared by mixing different kinds of oil with water. The separation efficiency was calculated based on:

(2) γ=m1m0×100%

where *m*0 and *m*1 are the masses of the liquid phase expelled before and after the separation process, respectively. As shown in Figure 4c, the oil flux decreased with the increase in mesh number of the stainless steel mesh, indicating a slower filtration rate but improved separation efficiency, because stainless steel meshes with higher mesh numbers were more effective in separating soybean oil/water mixtures, as shown in Figure 4d, with the 1000-mesh displaying the lowest oil flux (3800 L·m^−2^·h^−1^) and the highest separation efficiency (99.91%) which was better than the Graphene-Based Catalytic Membrane [56,57,58]. Prepared Graphene@PDMS-coated mesh, inspired by biomimetic structures, demonstrate the capability to efficiently and energy-efficiently separate oil/water mixtures and adjust the separation performance by altering the mesh number of the stainless steel mesh. An optimal balance between flux and separation efficiency was found with 400-mesh, as shown in Figure S15. That is the reason why we chose 400-mesh stainless steel mesh as the primary focus of our study. Based on the experiment shown in Figure 4b, we calculated that the wetting phase (heptane) permeated through the sample at a flux of approximately 27,400 L·m^−2^·h^−1^, while the non-wetting phase (water) was repelled above the MSSM, achieving an oil filtration rate exceeding 97%, with no contamination observed in the filtrate. In addition to heptane, the MSSM exhibited high durability towards other organic solvents and oils, including diesel, engine oil, soybean oil, and pump oil. The performance metrics for these substances are detailed in Figure 4e,f. Overall, for various oil-based emulsions, the modified hydrophobic coating mesh consistently exhibited a separation efficiency greater than 97%. Calculations revealed that for a range of oil/water mixtures, the MSSM consistently exhibited a separation efficiency greater than 97%. We also conducted the oil–water separation experiment in a vertical direction, as is shown in Movie S3.

## 4. Conclusions

The superhydrophobic coating was prepared by spraying the mixture of graphene and PDMS onto the substrate. Among them, a corrosion-resistant and robust superhydrophobic coating with a water CA greater than 150° was prepared on the 400-mesh stainless steel mesh. The simple one-step spraying approach is also applicable to other organic and inorganic substrates. Furthermore, the superhydrophobic coating maintains both chemical and physical stability, whether in harsh acidic or alkaline environments, as well as after ultrasonic treatment. Additionally, after soaking in a 5 wt% NaCl aqueous solution for 15 days, the surface morphology of the modified coating remained almost unchanged, confirming that the superhydrophobic properties were preserved. The 400-mesh modified stainless steel mesh effectively separates various oil/water mixtures, achieving a separation efficiency of over 97%, and the separation efficiency can be adjusted by changing the mesh size of the stainless steel mesh.

## Data Availability

All data generated or analyzed during this study are included in this published article and its Supplementary Materials.

## Supplementary Materials

The following supporting information can be downloaded at: https://www.mdpi.com/article/10.3390/biomimetics10050262/s1, Figure S1 The preparation of Graphene@PDMS coating on different materials. Figure S2 The image of the material (a) before and (c) after modification. The SEM image of nylon material (b) before and (d) after modification. Figure S3 The image of the material (a) before and (c) after modification. The SEM imgae of tissue material (b) before and (d) after modification. Figure S4 (a) The image of the material after modification. (b) The SEM imgae of 300 mesh stainless steel mesh after modification. Figure S5 The as-prepared PDMS@Graphene coating on non-woven fabric at (a) low and (b) high magnifications, respectively. Figure S6 The SEM image of the copper mesh after modification with different mesh sizes: (a) 200 mesh, (b) 300 mesh, (c) 400 mesh, (d) 500 mesh. The insert in each of them is the image of the corresponding material after modification. Figure S7 (a) Wetting behavior of strong acid, strong alkali, and water droplets on modified and original 400 mesh copper. (b) Wetting behavior of strong acid, strong alkali, and water droplets on modified and original 500 mesh copper. (c) Side view of strong acid, strong alkali, and water droplets on modified 400 mesh copper. (d) Side view of strong acid, strong alkali, and water droplets on modified 500 mesh copper. From the top down, the droplets will spread out into irregular shapes on the surface of copper mesh with virous mesh numbers before modification. While on the surface after superhydrophobic treatment, they are circular with similar size, and their upright state can be seen from the side; Figure S8 (a) Wetting behavior of strong acid, strong alkali, and water droplets on modified and original non-woven fabric. (b) Wetting behavior of strong acid, strong alkali, and water droplets on modified and original nylon cloth. (c) Side view of strong acid, strong alkali, and water droplets on modified non-woven fabric. (d) Side view of strong acid, strong alkali, and water droplets on modified nylon cloth. From the top down, the droplets will spread out into irregular shapes on the surface of non-woven fabric before modification and the phenomenom is strong on nylon cloth surface. While on the surface after superhydrophobic treatment, they are circular with similar size, and their upright state can be seen from the side. Figure S9 Wetting behavior of strong acid, strong alkali, and water droplets on (a) original and (b) modified weighting paper. (c) Side view of the droplets on modified weighting paper. From the top down, the droplets will spread out into irregular shapes on the surface of weighting paper before modification. While on the surface after superhydrophobic treatment, they are circular with similar size, and their upright state can be seen from the side. Figure S10 Wetting behavior of strong acid, strong alkali, and water droplets on (a) original and (b) modified printing paper. (c) Side view of the droplets on modified printing paper. From the top down, the droplets will spread out into irregular shapes on the surface of printing paper before modification. While on the surface after superhydrophobic treatment, they are circular with similar size, and their upright state can be seen from the side. Figure S11 Wetting behavior of strong acid, strong alkali, and water droplets on (a) original and (b) modified tissue. (c) Side view of the droplets on modified tissue. From the top down, the droplets will spread out into irregular shapes on the surface of tissue before modification. While on the surface after superhydrophobic treatment, they are circular with similar size, and their upright state can be seen from the side. Figure S12 (a) The contact angle of 200 mesh, 300 mesh, and 400 mesh stainless steel mesh before and after modification. (b) The contact angle of 200 mesh, 300 mesh, and 400 mesh copper mesh before and after modification. Figure S13 (a) The change of contact angle of water droplets on 500 mesh copper mesh after ultrasonic treatment. (b) The change of contact angle of water droplets on 500 mesh copper mesh after different pH treatments. Figure S14 Optical microscope images of (a) 300 mesh steel and (b) 300 mesh copper after ultrasonic treatment. Figure S15 The oil/water separation efficiency and oil flux versus the mesh numbers by taking the separation of soybean oil and water mixture as an example; Video S1 Droplet rolling experiment on nylon cloth; Video S2 Droplet rolling experiment on steel mesh; Video S3 Oil-water separation experiment in vertical direction.

## Abbreviations

The following abbreviations are used in this manuscript:

SSM	Stainless Steel Mesh
PDMS	Polydimethylsiloxane
GO	Graphene Oxide
MSSM	Modified Stainless Steel Mesh
OSSM	Original Stainless Steel Mesh
SEM	Scanning Electron Microscope
CA	Contact Angle
SSS	Static Superhydrophobic Surface
CBS	Cassie–Baxter State
pH	Acidity or Alkalinity Scale
NaCl	Sodium Chloride
HCl	Hydrochloric Acid
NaOH	Sodium Hydroxide
