# Supplementary material for: Lotus Leaf-Inspired Corrosion-Resistant and Robust Superhydrophobic Coating for Oil–Water Separation
Source: Biomimetics (Basel). 2025 Apr 24;10(5):262. doi: 10.3390/biomimetics10050262 (PMC12109249; doi:10.3390/biomimetics10050262)
Supplement: Supplementary file 1 [file biomimetics-10-00262-s001.zip › Superhydrophobic Coating-Supporting.pdf]

Supplementary Materials

Lotus Leaf-Inspired Corrosion-Resistant and Robust Superhydrophobic Coating for Oil-Water Separation

Wenhui Tu[1]†, Yiwen Luo[1]†, Junhao Shen[1], Xu Ran[1], Zhe Yu[1]*, Chaolun Wang[1], Chunhua Cai[1], and Hengchang Bi[1,2]*

[1] In Situ Devices Center, School of Integrated Circuit, East China Normal University, Shanghai 200241, China

[2] Chongqing Key Laboratory of Precision Optics, Chongqing Institute of East China Normal University, Chongqing 401120, China

* Correspondence: zyu@cee.ecnu.edu.cn (Z. Y.); hcbi@cee.ecnu.edu.cn (H. B.)

† These authors contributed equally to this work.

This PDF file includes:

Figures S1 to S15

Other Supplementary Materials for this manuscript include the following:

Movie S1 (mp4)

Droplet rolling experiment on steel mesh.

Movie S2 (mp4)

Droplet rolling experiment on nylon cloth.

Movie S3 (mp4)

Oil-water separation experiment in vertical direction.

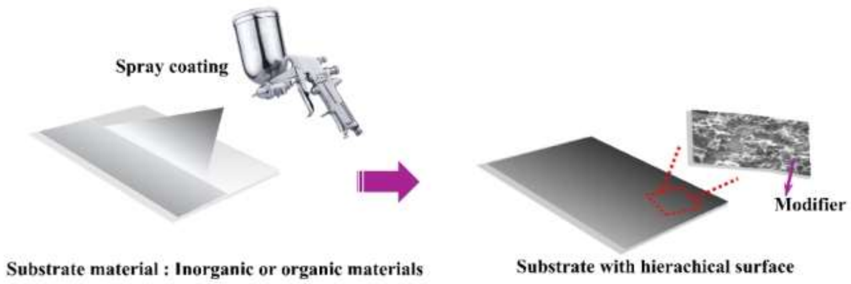

Figure S1. The preparation of Graphene@PDMS coating on different materials.

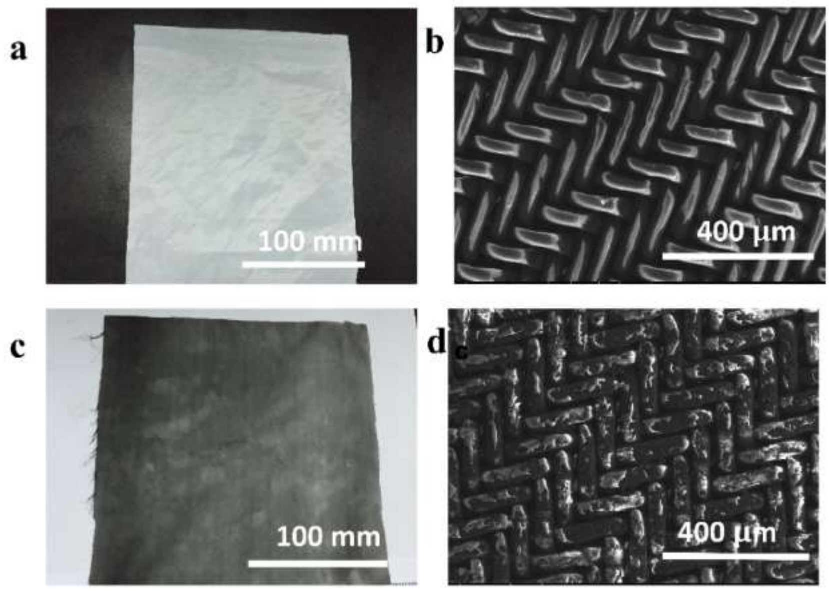

Figure S2. The image of the material (a) before and (c) after modification. The SEM image of nylon material (b) before and (d) after modification.

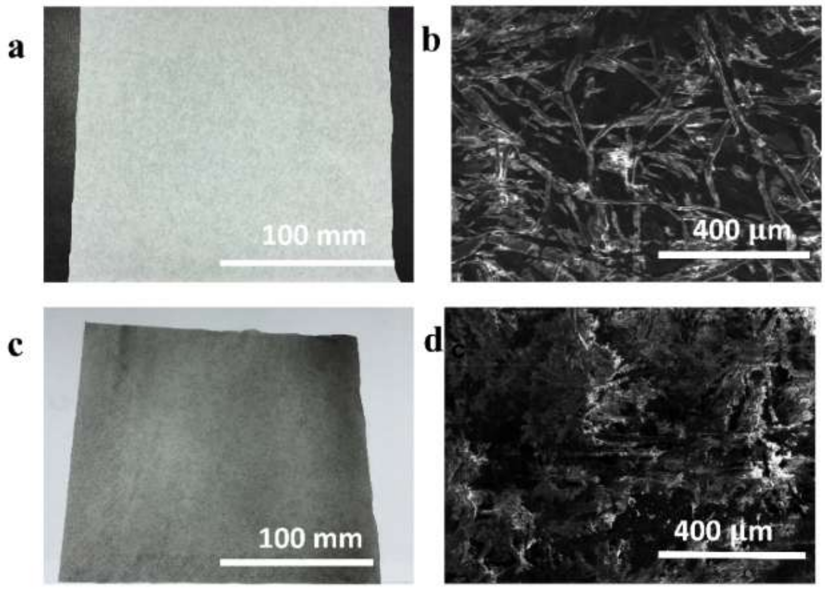

Figure S3. The image of the material (a) before and (c) after modification. The SEM imgae of tissue material (b) before and (d) after modification.

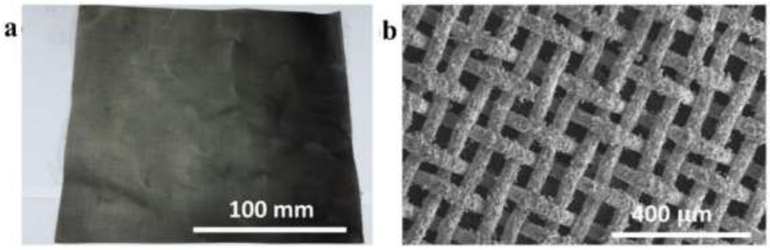

Figure S4. (a) The image of the material after modification. (b) The SEM imgae of 300 mesh stainless steel mesh after modification.

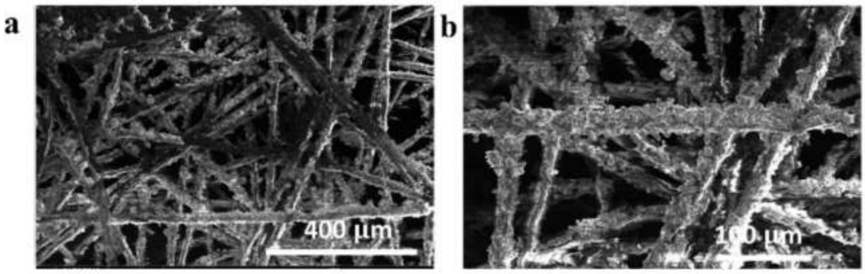

Figure S5. The as-prepared PDMS@Graphene coating on non-woven fabric at (a) low and (b) high magnifications, respectively.

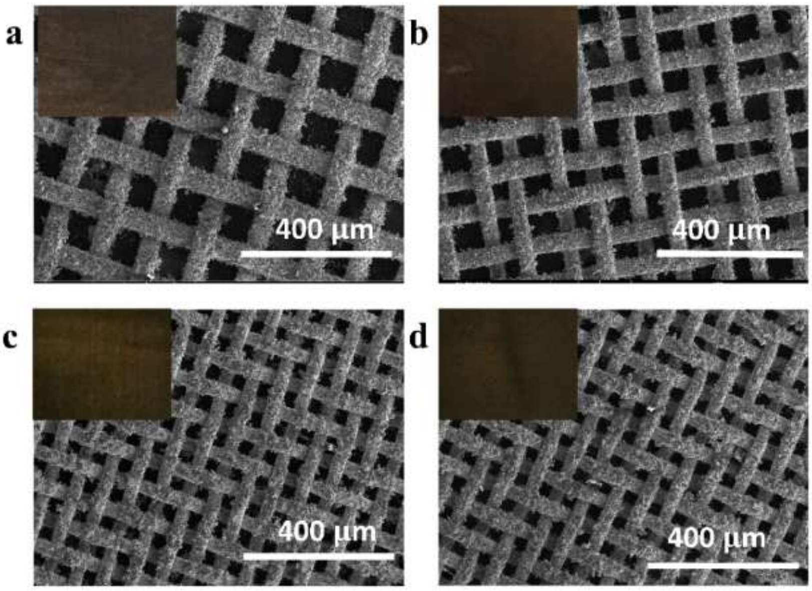

Figure S6. The SEM image of the copper mesh after modification with different mesh sizes: (a) 200 mesh, (b) 300 mesh, (c) 400 mesh, (d) 500 mesh. The insert in each of them is the image of the corresponding material after modification.

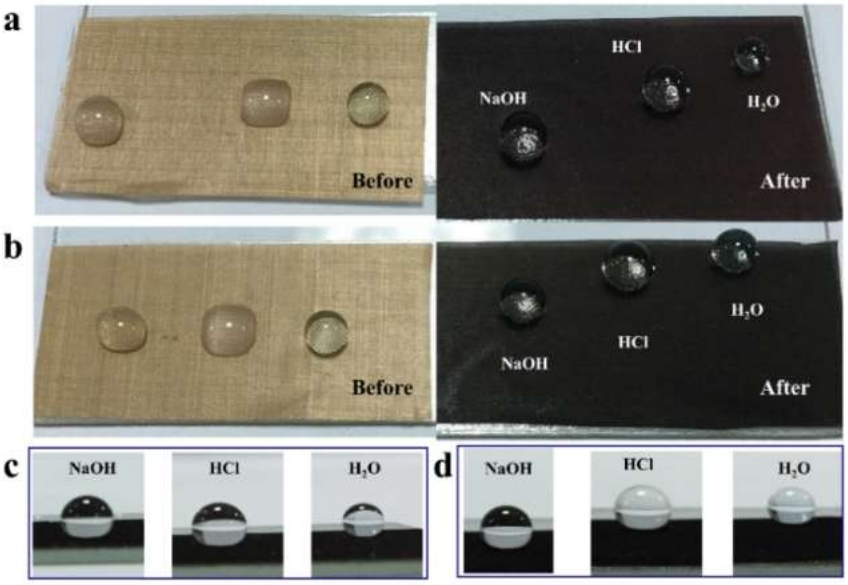

Figure S7. (a) Wetting behavior of strong acid, strong alkali, and water droplets on modified and original 400 mesh copper. (b) Wetting behavior of strong acid, strong alkali, and water droplets on modified and original 500 mesh copper. (c) Side view of strong acid, strong alkali, and water droplets on modified 400 mesh copper. (d) Side view of strong acid, strong alkali, and water droplets on modified 500 mesh copper.

From the top down, the droplets will spread out into irregular shapes on the surface of copper mesh with virous mesh numbers before modification. While on the surface after superhydrophobic treatment, they are circular with similar size, and their upright state can be seen from the side.

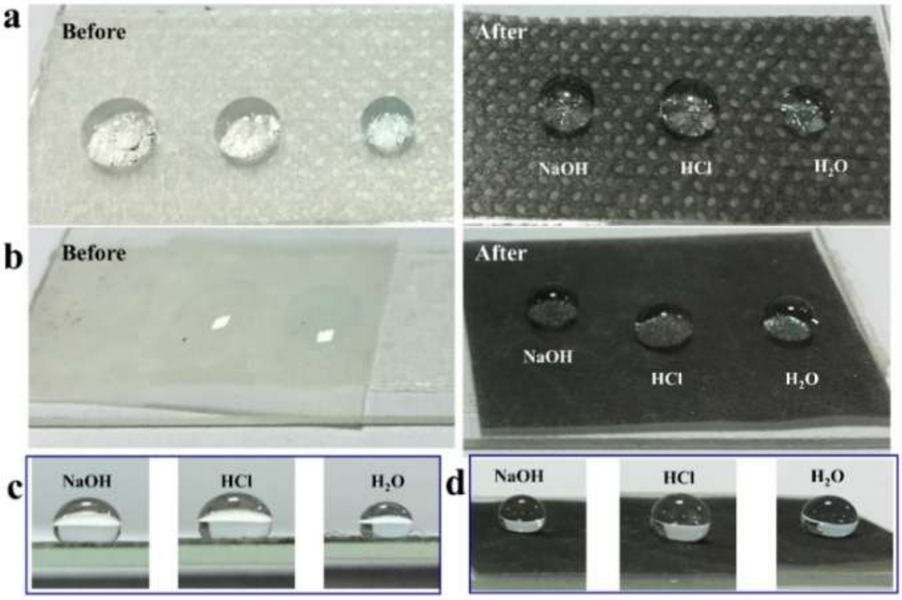

Figure S8. (a) Wetting behavior of strong acid, strong alkali, and water droplets on modified and original non-woven fabric. (b) Wetting behavior of strong acid, strong alkali, and water droplets on modified and original nylon cloth. (c) Side view of strong acid, strong alkali, and water droplets on modified non-woven fabric. (d) Side view of strong acid, strong alkali, and water droplets on modified nylon cloth.

From the top down, the droplets will spread out into irregular shapes on the surface of non-woven fabric before modification and the phenomenom is strong on nylon cloth surface. While on the surface after superhydrophobic treatment, they are circular with similar size, and their upright state can be seen from the side.

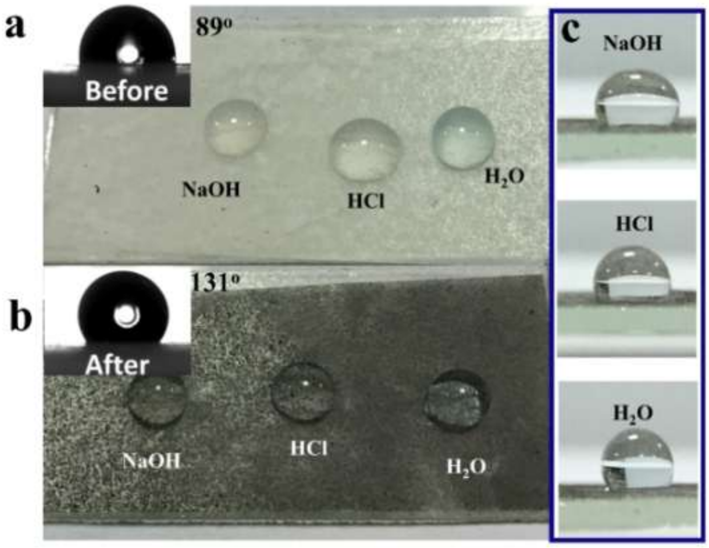

Figure S9. Wetting behavior of strong acid, strong alkali, and water droplets on (a) original and (b) modified weighting paper. (c) Side view of the droplets on modified weighting paper.

From the top down, the droplets will spread out into irregular shapes on the surface of weighting paper before modification. While on the surface after superhydrophobic treatment, they are circular with similar size, and their upright state can be seen from the side.

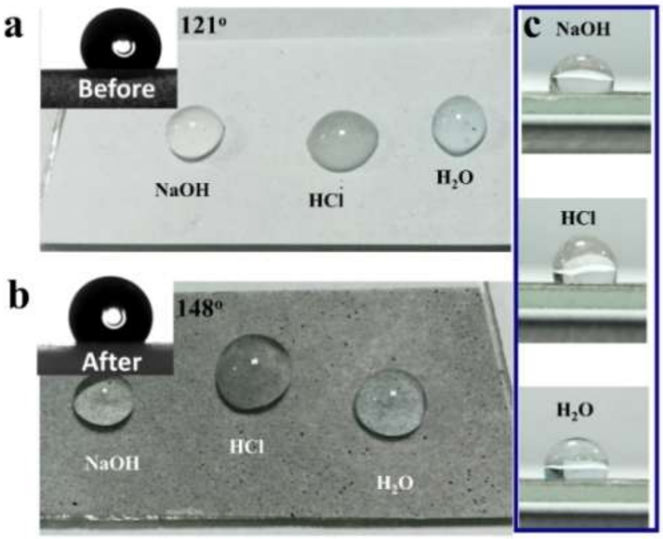

Figure S10. Wetting behavior of strong acid, strong alkali, and water droplets on (a) original and (b) modified printing paper. (c) Side view of the droplets on modified printing paper.

From the top down, the droplets will spread out into irregular shapes on the surface of printing paper before modification. While on the surface after superhydrophobic treatment, they are circular with similar size, and their upright state can be seen from the side.

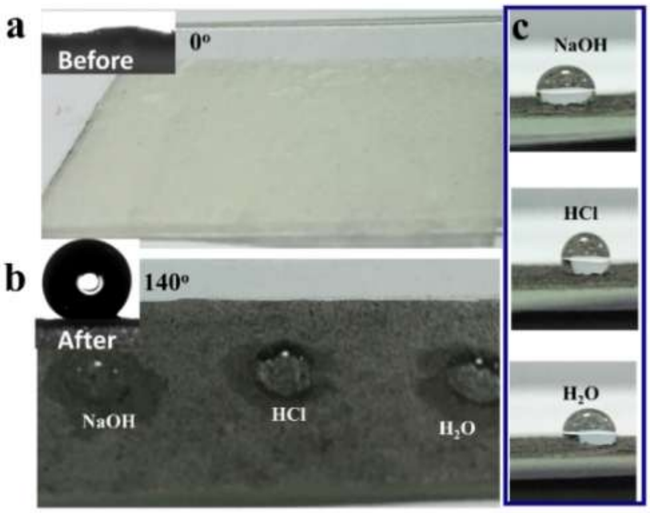

Figure S11. Wetting behavior of strong acid, strong alkali, and water droplets on (a) original and (b) modified tissue. (c) Side view of the droplets on modified tissue.

From the top down, the droplets will spread out into irregular shapes on the surface of tissue before modification. While on the surface after superhydrophobic treatment, they are circular with similar size, and their upright state can be seen from the side.

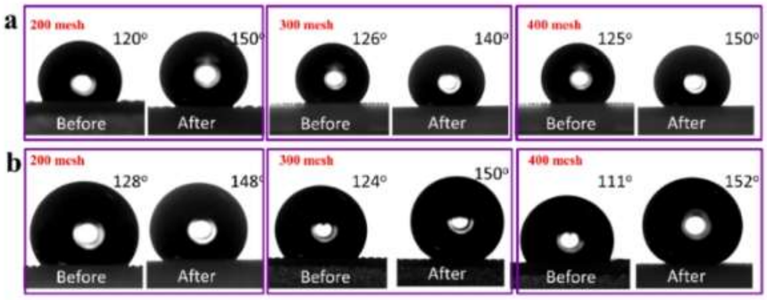

Figure S12. (a) The contact angle of 200 mesh, 300 mesh, and 400 mesh stainless steel mesh before and after modification. (b) The contact angle of 200 mesh, 300 mesh, and 400 mesh copper mesh before and after modification.

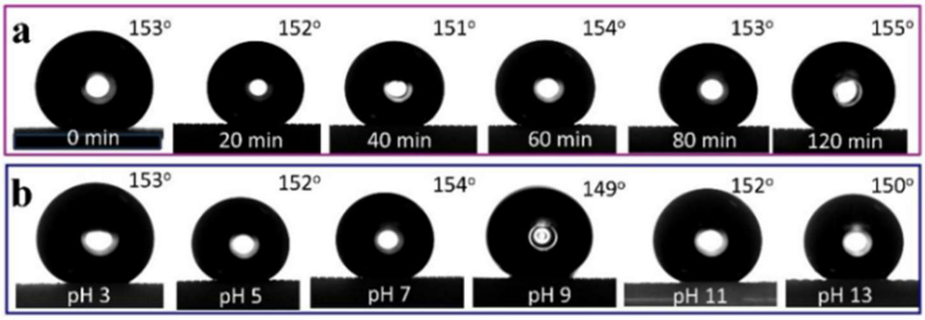

Figure S13. (a) The change of contact angle of water droplets on 500 mesh copper mesh after ultrasonic treatment. (b) The change of contact angle of water droplets on 500 mesh copper mesh after different pH treatments.

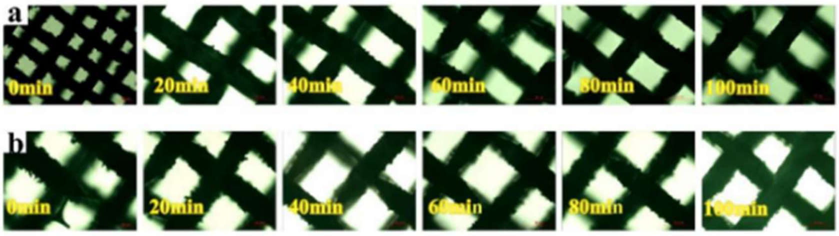

Figure S14. Optical microscope images of (a) 300 mesh steel and (b) 300 mesh copper after ultrasonic treatment.

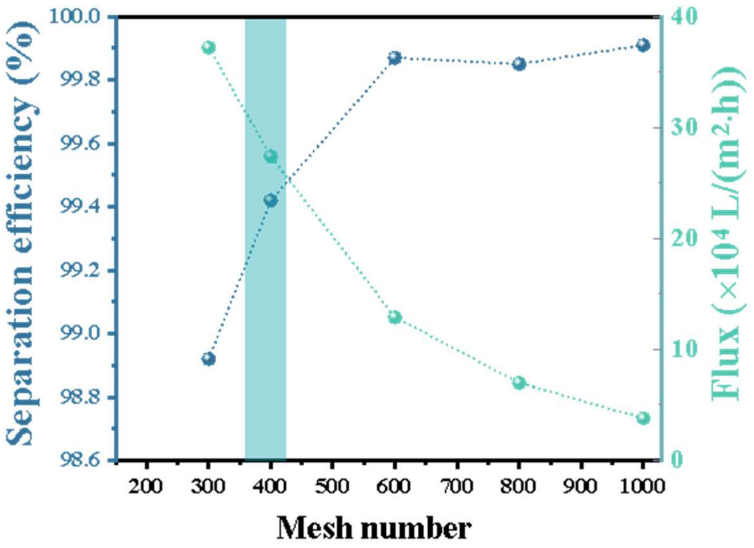

Figure S15. The oil/water separation efficiency and oil flux versus the mesh numbers by taking the separation of soybean oil and water mixture as an example.
